# The SMART App: an interactive web application for comprehensive DNA methylation analysis and visualization

**DOI:** 10.1186/s13072-019-0316-3

**Published:** 2019-12-05

**Authors:** Yin Li, Di Ge, Chunlai Lu

**Affiliations:** Department of Thoracic Surgery, Zhongshan Hospital, Fudan University, 180 Fenglin Road, Shanghai, 200032 People’s Republic of China

**Keywords:** TCGA, DNA methylation, Gene expression, Web application

## Abstract

**Background:**

Data mining of The Cancer Genome Atlas (TCGA) data has significantly facilitated cancer genome research and provided unprecedented opportunities for cancer researchers. However, existing web applications for DNA methylation analysis does not adequately address the need of experimental biologists, and many additional functions are often required.

**Results:**

To facilitate DNA methylation analysis, we present the SMART (Shiny Methylation Analysis Resource Tool) App, a user-friendly and easy-to-use web application for comprehensively analyzing the DNA methylation data of TCGA project. The SMART App integrates multi-omics and clinical data with DNA methylation and provides key interactive and customized functions including CpG visualization, pan-cancer methylation profile, differential methylation analysis, correlation analysis and survival analysis for users to analyze the DNA methylation in diverse cancer types in a multi-dimensional manner.

**Conclusion:**

The SMART App serves as a new approach for users, especially wet-bench scientists with no programming background, to analyze the scientific big data and facilitate data mining. The SMART App is available at http://www.bioinfo-zs.com/smartapp.

## Introduction

All cancers arise as a result of the accumulation of somatic mutations, copy number alterations, and epigenetic modifications that alter transcription and protein expression. Thus, studies of molecular features such as DNA methylation may reveal the underlying mechanisms of carcinogenesis and progression. DNA methylation, the addition of a methyl group to DNA, plays a critical role in regulating gene expression [1]. It has been reported that DNA methylation at the promoter regions is often negatively correlated with gene expression while DNA methylation in gene bodies is often positively correlated with gene expression [2]. Abnormal DNA methylation patterns are found in every type of human cancer [3]. Many previous studies have shown that DNA methylation is involved in many aspects of carcinogenesis and provides potential biomarkers for evaluating the diagnosis and prognosis of cancer [4–6]. A recent study has also shown the association between DNA methylation and somatic copy number aberration, suggesting a much more complex mechanism beyond this modification [7].

The Cancer Genome Atlas (TCGA), a project supported by the National Cancer Institute (NCI) and National Human Genome Research Institute (NHGRI), hosts tremendous amount of multi-omics data and allows systematic study of the genetic or epigenetic basis of cancer [8]. However, accessing and analyzing the DNA methylation data from TCGA database is quite difficult for those scientists who have no computational background. Therefore, constructing easy-to-use applications for analyzing the DNA methylation data of TCGA database is demanded.

MethHC (http://methhc.mbc.nctu.edu.tw), Wanderer (http://maplab.imppc.org/wanderer/), MEXPRESS (https://mexpress.be), and MethSurv (https://biit.cs.ut.ee/methsurv/) are examples of web-based tools that allow researchers to integrate, analyze, and visualize DNA methylation [9–12]. MethHC enables users to browse the top 250 hyper- or hypo-methylated genes in 18 cancer types. Wanderer allows users to analyze DNA methylation and gene expression in a regional framework, MEXPRESS allows users to look at DNA methylation data in relation to its genomic location, and MethSurv can associate overall cancer survival with DNA methylation levels across a large body of TCGA data and many cancers. Although these tools are exceptionally valuable, they do not fully unlock the potential of the publicly available data. For example, they do not offer a function for users to explore the correlation between DNA methylation and transcript expression. In addition, none of the above tools help users visualize the chromosomal distribution of differential methylated CpGs in diverse cancer types. Therefore, we developed the SMART App, which enables users to analyze DNA methylation and its association with other omics data. The SMART App can facilitate DNA methylation data mining and help reveal the complexity of epigenetic modifications.

## Results

### Features

The SMART App offers interactive functions for users to analyze the DNA methylation in diverse cancer types in a multi-dimensional way.

#### Home

**Fig. 1:**
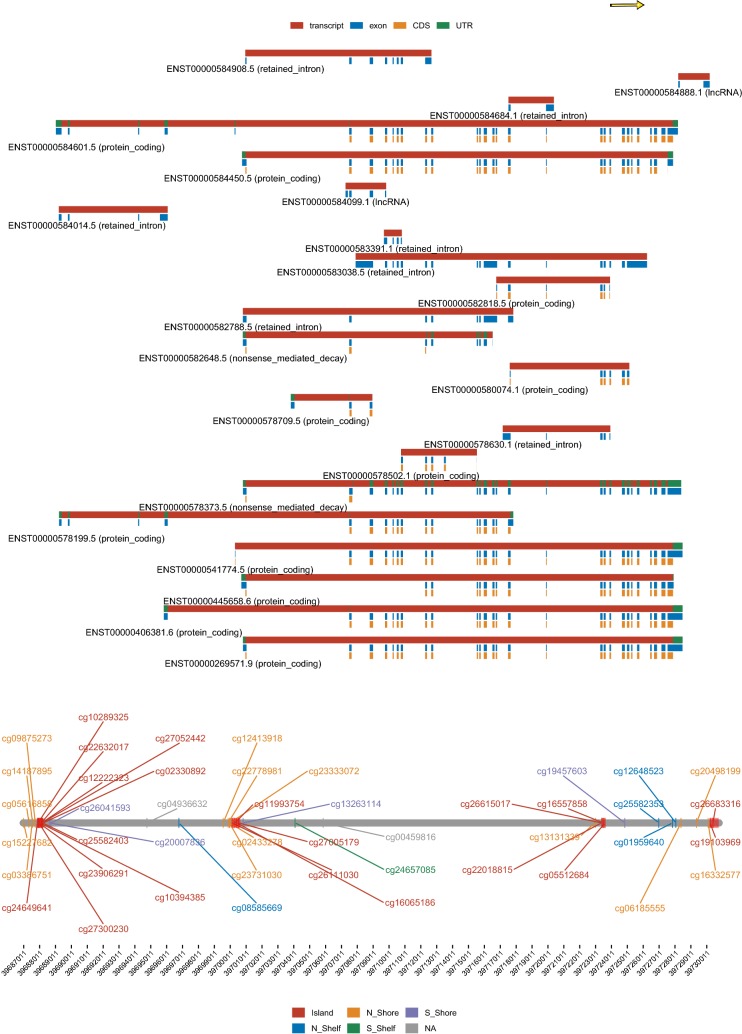
Genomic information of the gene ERBB2. The segment plot showing the detailed information of genomic locations of CpGs of ERBB2, highlighting transcripts, exons, UTR, CDS (coding sequence), CpG island, shelves, and shores. The name and the type of each transcript are given. The genomic length is shown below. By default, the distance between any adjacent two lines stands for 1 k. Users can set the distance scale. The yellow arrow at the top stands for the strand direction, that is, towards right, +, towards left, −. The coverage of the CpG islands are displayed as the red regions

The home page first displays the number of DNA methylation samples available from TCGA project, colored by sample types (i.e., Normal and Tumor), for users to gain an overview of the sample size of the cancer type of interest. Next, the SMART App provides a quick search interface. Users can enter a gene symbol (e.g., ERBB2) into the ‘Quick start’ box to search for a gene of interest. By clicking the “Go” button, a circular plot showing the chromosomal distribution of all associated CpGs of the input gene will be displayed. To help users gain more useful information about the CpGs and their genomic locations along with transcripts, a detailed segment plot highlighting the transcripts, exons, UTR, CDS, CpG island regions, shelves and shores is displayed below (Fig. 1). This segment plot can help researchers to identify potential methylation-expression related CpGs. The panel below summarizes the detailed information these probes, and users can select one of these probes to view its pan-cancer methylation profile and identify aberrantly methylated sites for further analysis. Besides, users can also view the CpG-aggregated pan-cancer methylation profile. Users can select multiple CpGs at a time to explore the mean or median methylation of the selected CpGs. We previously identified that TRIM58 is a novel prognostic-related methylation-driven gene in lung squamous cell carcinoma [13]. Using the quick search function of the SMART App, it is easy to find that mean methylation level of TRIM58 is significantly higher not only in lung squamous cell carcinoma but also in many other cancer types including breast cancer, head and neck carcinoma, and lung adenocarcinoma, indicating its potential role in carcinogenesis in these cancer types.

#### Differential CpGs

**Fig. 2:**
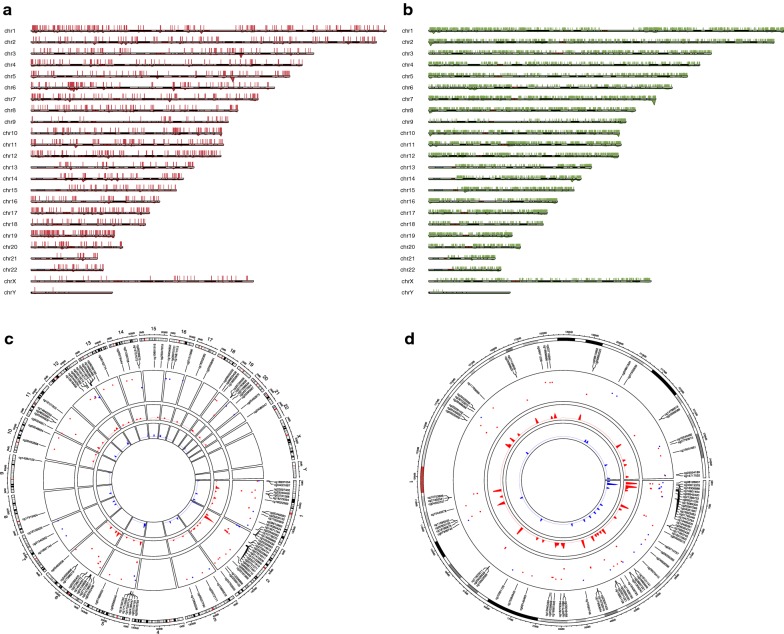
Visualization of the differentially methylated CpGs in BLCA with delta |Beta-value| > 0.25 and *p* value < 0.01. **a** There are 6007 hyper-methylated CpGs in BLCA, with chromosome 1, 19 containing the highest number of hyper-methylated CpGs, and chromosome Y containing the least. **b** There are 23,249 hypo-methylated CpGs in BLCA. **c** Custom chromosome plot showing the selected hyper- and hypo-methylated CpGs on all chromosomes. **d** Custom chromosome plot showing the selected CpGs on chromosome 1

Differential analysis is a common approach in cancer research by comparing tumor samples vs. normal samples for identifying aberrantly methylated CpGs. Meanwhile, clustering of the CpGs with similar methylation patterns along the chromosomes may reflect the genomic mechanisms leading to specific methylation characteristics [14]. Therefore, the SMART App allows users to set custom cut-off values for a given cancer type to dynamically obtain differentially methylated CpGs and their chromosomal distributions (Fig. 2). The delta |Beta-value|/delta |*M* value| of each probe is calculated as the mean Beta-value/*M* value in tumor samples minus the mean Beta-value/*M* value in normal samples. *p* value is calculated using the Wilcoxon rank sum test, and is adjusted using the Benjamini–Hochberg method. Moreover, for users who only want to visualize specific CpGs, the SMART App offers an extra function that allows users to draw CpG flexibly. The detailed description can be found at the website.

#### Methylation DIY

**Fig. 3:**
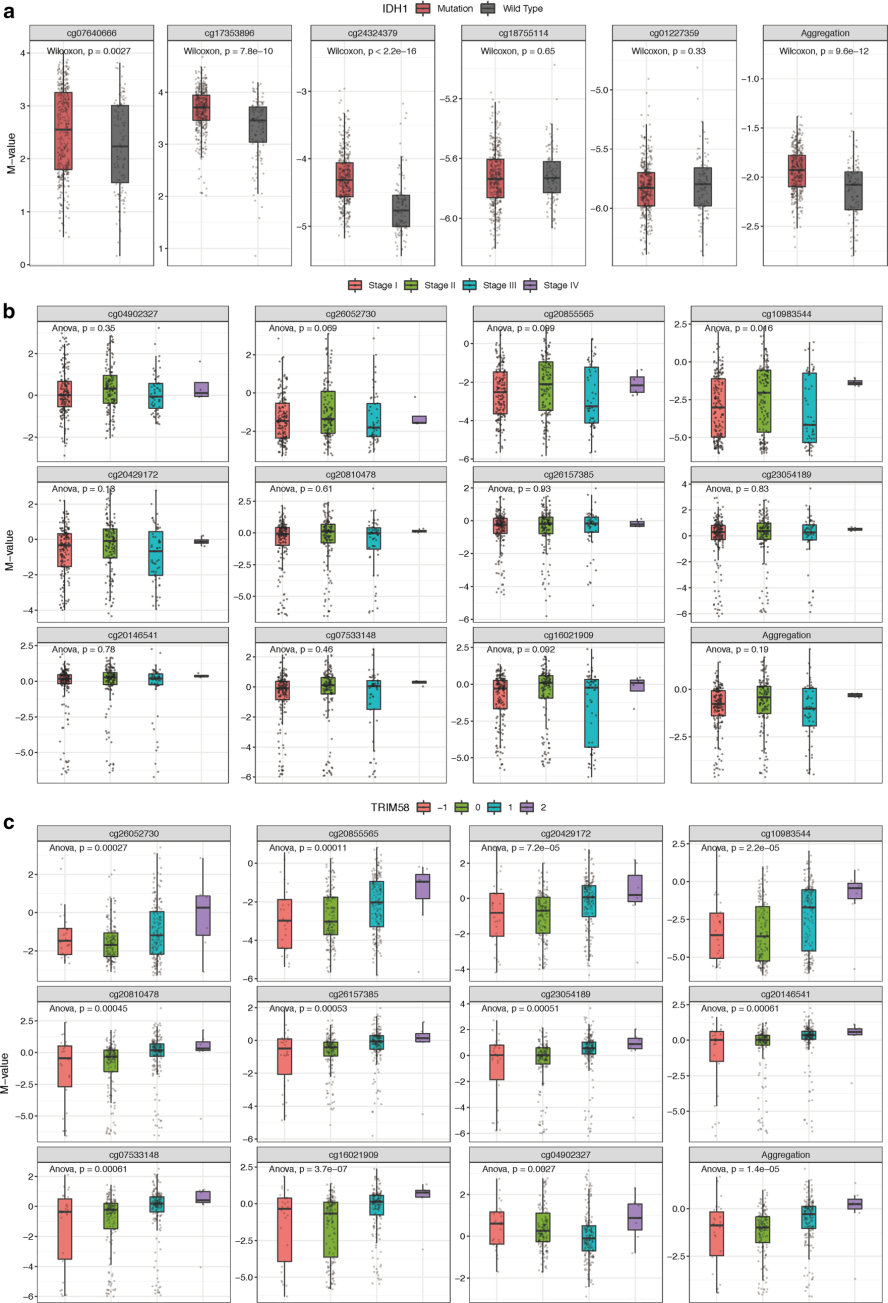
Methylation DIY. **a** cg07640666, cg17353896, and cg24324379 are hyper-methylated in IDH1 mutation group in LGG (*M* value, *p* value < 0.05). **b** Major stage plot showing cg10983544 (TRIM58) is much hyper-methylated in stage II lung squamous cell carcinoma (*M* value, *p* value = 0.016). **c** cg04902327 (TRIM58) shows a lower level of methylation with low-level copy number amplification, whereas the other CpGs of TRIM58 show a positive correlation with CNV in lung squamous cell carcinoma (*M* value, *p* value < 0.05). − 2: homozygous deletion; − 1: single copy deletion; 0: diploid normal copy; + 1: low-level copy number amplification; + 2: high-level copy number amplification

This module provides functions for users to comprehensively analyze DNA methylation taking other omics data and clinical stages into consideration. The first panel generates custom box plots for users for compare CpGs of genes between normal and tumor samples in a given cancer type. Users can select multiple probes at the same time for easy visibility and interpretation. The returned box plots will display all the selected probes plus an aggregation box plot showing the mean/median methylation of all the selected probes. The second panel plots methylation by pathological stages based on the TCGA clinical data. Two options are available, namely, major stage and sub-stage. For example, if users choose major stage for plotting, stage IIA/IIB will be included in the stage II group. Here, using SMART App, we can easily find that TRIM58 (cg10983544) is much hyper-methylated in stage II group in lung squamous cell carcinoma, indicating its clinical relevance (Fig. 3b, *p* value = 0.016). Somatic mutations can also affect DNA methylation. To help users study the effect of somatic mutations on DNA methylation, the SMART App offers a function for plotting box plots comparing methylation between mutation and wild-type groups. For example, IDH1 mutation can cause hyper-methylation in lower grade glioma (LGG) [15]. When IDH1 is selected, the returned box plots showed that cg07640666, cg17353896 and cg24324379 were significantly hyper-methylated in the mutation group (Fig. 3a, *p* value < 0.05). Sun et al. observed the correlation between CNV and methylation and discussed the possible mechanisms relating to this event [7]. Here, the SMART App provides a function for researchers to study the possible association between CNV and DNA methylation. The results are displayed as box plots showing the correlation between CNV and methylation. With the SMART App, it is very interesting to observe that TRIM58 (cg04902327) shows a lower level of methylation with low-level copy number amplification, whereas other CpGs of TRIM58 show a positive correlation with CNV in lung squamous cell carcinoma (Fig. 3c, *p* value < 0.05).

#### Correlation

**Fig. 4:**
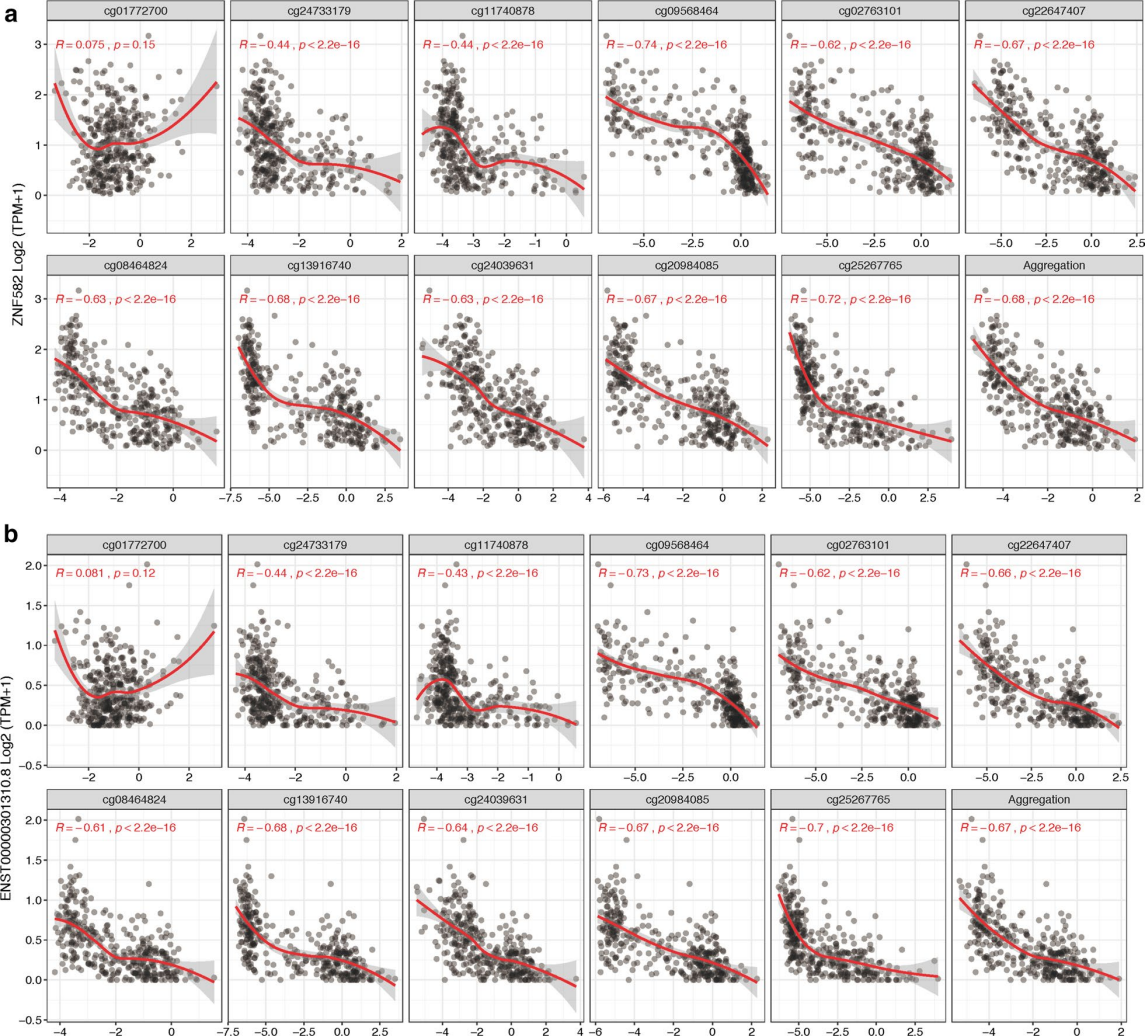
Spearman correlation between expression (ZNF582) and DNA methylation (*M* value) in lung squamous cell carcinoma. **a** Gene-level correlation showing that the expression of ZNF582 is significantly negatively associated with the methylation of cg24733179, cg11740878, cg09568464, cg02763101, cg22647407, cg08464824, cg13916740, cg24039631, cg20984085, and cg25267765. **b** Transcript-level correlation showing that the expression of ENST00000301310.8 is significantly negatively associated with the methylation of cg24733179, cg11740878, cg09568464, cg02763101, cg22647407, cg08464824, cg13916740, cg24039631, cg20984085, and cg25267765

DNA methylation is often correlated with gene expression. The correlation function of SMART App performs correlation analysis between gene expression and methylation for any given sets of TCGA, using methods including Pearson, Spearman, and Kendall correlation statistics. The UCSC Xena provides the re-computed expression data of TCGA for 198,619 transcripts. Accordingly, there are two levels available, and one can choose to analyze the correlation at gene level or transcript level. When analyzing the correlation at transcript level, a segment plot highlighting the genomic locations of the transcript and CpGs will be displayed, and the distances of each probe to TSS will also be shown in the table below for users to locate the ones at the promoter region. The results are displayed as scatter and distribution plots (Fig. 4 and Additional file 1: Figure S1).

#### Survival

The SMART App performs overall survival (OS) and disease-free internal (DFI)-related survival analysis based on methylation levels. This function allows users to select their custom cancer types for overall or disease-free survival analysis. Cox regression analysis is a popular method for evaluating the prognostic value of individual variables. To efficiently analyze the survival significance of methylation, the SMART App offers both univariate and multivariate Cox regression analyses. When performing multivariate Cox regression analysis, users can adjust for potential confounding factors, including age, gender, race and pathological stage. Users can copy and paste a list of CpGs into the box, and select the cancer type of interest to conduct Cox regression analysis. The hazard ratio, 95% confidence interval, *z* score, and *p* value will be given. Once users have identified the significant variables, they can use the SMART App to draw survival curves. The thresholds for high/low methylation level cohorts can be adjusted by users.

### Comparison with existing tools

Web tools to analyze DNA methylation of TCGA project include methHC, Wanderer, MEXPRESS, and MethSurv. MethHC was introduced in 2014 and enables users to identify highest/lowest methylated genes, perform hierarchical cluster analysis, explore methylation profile across tumors and conduct correlation analysis. However, the latest update of methHC was in 2014. Wanderer is an interactive web application to explore DNA methylation and gene expression. It provides a single-page interface to explore DNA methylation in a regional framework. MEXPRESS is a data visualization tool for DNA methylation analysis and was first introduced in 2015. Now, it has been updated, adding more data and generating fancier figures. MethSurv is a shiny application that mainly focuses on the clinical impacts of DNA methylation. While these tools are extraordinarily valuable, many extra functions are not adequately addressed by them. *M* value has been reported to be more statistically valid for the differential analysis [16]. Although differential analyses are commonly performed by these tools, none of them allow users to use the *M* value for differential analysis. None of these tools allow users to pick a cancer type and visualize the chromosomal distribution of the aberrantly methylated CpGs. In addition, none of the existing tools allow users to analyze the correlation between methylation and expression at transcript level. Besides, none of the tools provide customizable selection of methylation thresholds for patient cohort partitioning in survival curves plotting. A detailed comparison is shown in Table 1.

**Table 1:** Functionalities comparison between the SMART App and other tools

	SMART App	methHC	Wanderer	MEXPRESS	MethSurv
Data source	TCGA	TCGA	TCGA	TCGA	TCGA
Latest update	2019	2014	2018	2019	2018
Genomic location visualization	Yes	No	Yes	Yes	No
Pan-cancer methylation profile	Yes	Yes	No	No	No
Differential analysis	Yes	Yes	Yes	Yes	Yes
Correlation with other omics data	Yes	No	No	Yes	No
Correlation with gene expression	Yes	Yes	Yes	Yes	Yes
Correlation with transcript expression	Yes	No	No	No	No
Cox regression analysis	Yes	No	No	No	Yes
Survival analysis with custom threshold	Yes	No	No	No	Yes
Hierarchical cluster analysis	No	Yes	No	No	Yes
Methylation *M* value	Yes	No	No	No	No

A yes means this function is available

## Discussion

The SMART App is an interactive web application for DNA methylation analysis based on the TCGA database. The SMART App enables experimental biologists without any computational programming background to perform various analyses relating to DNA methylation in diverse cancer types. Using the SMART App, one can easily explore the large DNA methylation data, ask specific scientific questions, and validate their findings. For example, one can easily find that CpGs such as cg10983544 and cg20429172 are located at the promoter region of the transcript of TRIM58, and may ask whether these CpGs are aberrantly methylated and whether the methylation changes of these CpGs will lead to gene expression alterations. One can also identify significantly hyper- and hypo-methylated CpG-based custom thresholds. Moreover, one can explore the correlation between methylation and other omics and clinical data, analyze the prognostic value of CpGs and draw survival curves. Meanwhile, the flexible customization parameters of the SMART App also enable users to customize the result visualization. The SMART App is a user-friendly and intuitive tool for unlocking the potential value of the genomic data in TCGA. It complements well with other available tools.

## Conclusion

The SMART App is a web-based tool to explore and interpret the DNA methylation data across 33 cancer types from TCGA database. The source code of the SMART App is available for users to download under GPLv3 license.

## Methods

The SMART App is developed entirely in the R programming language using the Shiny framework and is freely available for all users. There is no login requirement for accessing any features in the SMART App. The SMART App has been most extensively tested in a Safari browser environment and is also compatible with other popular web browsers such as Chrome, Firefox, and Internet Explorer.

The data used in the SMART App are directly pulled down from the TCGA Pan-Cancer cohort of UCSC Xena public data hubs (https://xenabrowser.net) upon users’ request using *UCSCXenaTools*, including gene expression (TOIL re-computed TPM), transcript expression (TOIL re-computed TPM) [17], DNA methylation (HumanMethylation450k; Primary Solid Tumor and Solid Normal Tissue), somatic mutation (Gene-level non-silent mutation), copy number variation (Gene-level GISTIC2 thresholded), phenotype and clinical information [18]. Gene-level non-silent mutation and gene-level thresholded copy number variation are used because they offer easy-to-interpret values (i.e., 0 for wild type and 1 for mutation for somatic mutation; homozygous deletion (− 2), single copy deletion (− 1), diploid normal copy (0), low-level copy number amplification (+ 1) and high-level copy number amplification (+ 2) for copy number variation). For methylation probes, we used the hg38 coordinates provided by Zhou et al. (http://zwdzwd.github.io/InfiniumAnnotation) [19]. Gene, transcript and exon coordinates were obtained from GENCODE (https://www.gencodegenes.org, Release 31, GRCh38.p12).

Both Beta-value and *M* value are commonly used in DNA methylation analysis. The *M* value has been reported to have a more dynamic range, and is more appropriate for statistical analysis [16]. Whereas the Beta-value is much more biologically interpretable. Therefore, these two types of methylation values are available in the SMART App.

The SMART App outputs consist of figures and tables, which are available for users to download. Figures are rendered as Portable Document Format (PDF), which can be further edited using Adobe Illustrator. Tables are generated by *DT* R package (https://rstudio.github.io/DT/) allowing for data querying and selection.

## Supplementary information

**Additional file 1: Figure S1.** Distribution plots showing the correlation between expression and Methylation. Each bar represents a sample, the names of the gene/transcript and CpGs are shown on the right, the methylation and expression values are shown on the left. The samples are reorders according to the expression value. A. Gene-level distribution plot. B. Transcript-level distribution plot.

## Data Availability

Not applicable.

## Abbreviations

TCGA: The Cancer Genome Atlas
SMART: Shiny Methylation Analysis Resource Tool
CNV: copy number variation
