# Supplementary material for: The SMART App: an interactive web application for comprehensive DNA methylation analysis and visualization
Source: Epigenetics Chromatin. 2019 Dec 5;12:71. doi: 10.1186/s13072-019-0316-3 (PMC6894252; doi:10.1186/s13072-019-0316-3)
Supplement: Supplementary file 1 — Additional file 1: Figure S1. Distribution plots showing the correlation between expression and Methylation. Each bar represents a sample, the names of the gene/transcript and CpGs are shown on the right, the methylation and expression values are shown on the left. The samples are reorders according to the expression value. A. Gene-level distribution plot. B. Transcript-level distribution plot. [file 13072_2019_316_MOESM1_ESM.docx]

**Additional information**

**The SMART App: an interactive web application for comprehensive DNA methylation analysis and visualization**

Yin Li, Di Ge, and Chunlai Lu^*^

Department of Thoracic Surgery, Zhongshan Hospital, Fudan University, Shanghai, P. R. China.

^*^Corresponding author: Chunlai Lu, M.D. Department of Thoracic Surgery, Zhongshan Hospital, Fudan University, 180 Fenglin Road, Shanghai, 200032, P.R.China, Tel. /Fax: +86 021 64041990-2559, E-mail address: lu.chunlai@zs-hospital.sh.cn.

**Running title**: an interactive web application for DNA methylation

**Figure S1. Distribution plots showing the correlation between expression and Methylation.** Each bar represents a sample, the names of the gene/transcript and CpGs are shown on the right, the methylation and expression values are shown on the left. The samples are reorders according to the expression value. A. Gene-level distribution plot. B. Transcript-level distribution plot.
